# Evolution of Orexin Neuropeptide System: Structure and Function

**DOI:** 10.3389/fnins.2020.00691

**Published:** 2020-07-10

**Authors:** Shingo Soya, Takeshi Sakurai

**Affiliations:** Faculty of Medicine/International Institute for Integrative Sleep Medicine (WPI-IIIS), University of Tsukuba, Tsukuba, Japan

**Keywords:** neuropeptide, orexin, OX1R, OX2R, hypothalamus, vertebrate

## Abstract

Orexins are hypothalamic neuropeptides that were initially identified in the rat brain as endogenous ligands for an (previously) orphan G-protein-coupled receptor (GPCR). They are multitasking peptides involved in many physiological functions, including regulation of feeding behavior, wakefulness and autonomic/neuroendocrine functions, and sleep/wakefulness states in mammals. There are two isopeptides of orexin, orexin A and orexin B, which are produced from a common precursor peptide, prepro-orexin. Structures of orexins, as well as orexin genes, are highly conserved throughout mammalian species, suggesting strong evolutionary pressure that maintains the structures. Their lengths and structure suggested that orexin B is the ancestral form of the orexin neuropeptide. In mammals, orexins bind to two subtypes of GPCRs, i.e., orexin 1 receptor (OX1R) and orexin 2 receptor (OX2R). Phylogenetically, the orexin system is present exclusively in vertebrates. In genomes of species outside mammals, there is only one orexin receptor, which is similar to OX2R, suggesting that OX2R is the prototype receptor for orexins. OX1R is likely to have evolved during early mammalian evolution. Orexin-producing neurons (orexin neurons) are mainly located in the lateral hypothalamic area (LHA) in mammals and are also found in hypothalamic regions in many other vertebrates. Orexins are likely to be closely related to the regulation of active, motivated behavior in many species. The orexin system seems to have evolved as a system that supports active and purposeful behavior which is closely related with wakefulness.

## Introduction

Orexins were initially recognized as regulators of feeding behavior. Subsequently, the finding that orexin deficiency causes narcolepsy in several mammalian species revealed that orexins play a critical role in regulation of sleep/wakefulness states, especially in maintenance of wakefulness in mammals. Orexins were also shown to be involved in the regulation of a wide range of physiological functions, suggesting that orexins are multitasking peptides. Any purposeful behavior requires certain internal body states, including appropriate tuning of the autonomic nervous system and endocrine function. Maintenance of wakefulness and vigilance is also important for pursuing behaviors, because appropriate arousal levels are especially necessary for executing any purposeful behavior that requires high motivation. The systems involved in these functions are closely related and are interconnected with the orexin system (Sakurai, 2014). Orexin-producing neurons (orexin neurons), which locate in the LHA, receive input by forebrain structures including the extended amygdala and nucleus accumbens (NAc)—which are implicated in the processing of emotion and motivation—and send output to brain stem regions, which are implicated in the regulation of wakefulness. Orexin neurons play an important role as a link between emotional states and wakefulness states.

In non-mammalian species, sleep/wakefulness states are generally solely defined by behavioral criteria, and wakefulness is defined as a state with active behavior. Orexins are likely to play an important role in regulation of active behavior also in non-mammalian species, and these factors are also recognized as regulators of wakefulness. This review focuses on how the structures of orexins and their receptors, neuronal circuits, and their functions have evolved in the animal kingdom.

## Summary of the Mammalian Orexin System

The hypothalamus plays a central role in the integrated control of feeding and energy homeostasis. We identified two novel neuropeptides, both derived from the same precursor by proteolytic processing, that bind and activate two closely related previously orphan GPCRs. These peptides, termed orexins A and B, had no significant structural similarities to known families of regulatory peptides (Sakurai et al., 1998). *Prepro-orexin* mRNA and immunoreactive orexin are specifically localized in neurons within and around the lateral and posterior hypothalamus in the adult rat brain. When administered centrally to rats, these peptides increased food consumption. *Prepro-orexin* mRNA level is upregulated by fasting, suggesting a physiological role of these peptides as mediators in the central feedback mechanism that regulates feeding behavior (Sakurai et al., 1998). Molecular cloning studies showed that orexins A and B are derived from a common precursor peptide, prepro-orexin. An mRNA encoding the same precursor peptide was independently identified by De Lecea et al. (1998) as a hypothalamus-specific transcript. The authors predicted that the transcript encoded a polypeptide precursor that is cleaved to form two neuropeptides, termed hypocretin-1 and hypocretin-2 (corresponding to orexins A and B, respectively).

Our structural analysis of the purified peptides revealed that orexin A is a 33-amino-acid peptide with an N-terminal pyroglutamyl residue, two intrachain disulfide bonds, and C-terminal amidation. Strikingly, this structure is completely conserved among all mammalian species so far identified (human, gorilla, rat, mouse, cow, pig, sheep, dog, seal, and dolphin). Mammalian orexin B is a 28-amino-acid, C-terminally amidated linear peptide, which also has a highly conserved structure among mammalian species. The C-terminal half of orexin B is very similar to that of orexin A, whereas the N-terminal half is more variable (Sakurai et al., 1998) (Figure 2). The unusually conserved structures of orexins suggest strong evolutionary pressure that preserves the structure, which is likely to be related with the function of these peptides.

The best understood role of orexins in mammals is regulation of sleep and wakefulness states, especially in the maintenance of long, consolidated wakefulness. This is highlighted by findings that orexin deficiency caused narcolepsy in several mammalian species including mice, rats, dogs, and humans (Chemelli et al., 1999; Lin et al., 1999; Peyron et al., 2000; Thannickal et al., 2000; Sakurai, 2007). Sleep and wakefulness are regulated to occur at appropriate times, in accordance with the internal and external environments. Avoiding danger and finding food, which are life-essential activities that are regulated by emotion, reward, and energy balance, require vigilance and therefore, by definition, wakefulness. The orexin system is involved in these functions (Sakurai et al., 1998; Yamanaka et al., 2003). Other than that, orexin has been implicated in a variety of functions including regulation of food intake, emotion, the reward system, and the autonomic nervous system. These functions of orexins are mediated by two GPCRs, OX1R and OX2R. OX1R has a greater affinity for orexin A over orexin B, whereas OX2R binds both ligands with similar affinities. Orexin receptors exhibit a markedly different distribution. They are abundantly expressed by monoaminergic neurons in the brain stem (Mieda et al., 2011). Orexin neurons, which have been assumed to number around 3,000 in the rat brain and around 70,000 in the human brain, are localized exclusively in the hypothalamus, including the LHA, perifornical area, and posterior hypothalamus. These neurons send widespread projections to the brain, with particularly dense projections to monoaminergic and cholinergic nuclei in the brain stem, where OX1R and OX2R are differentially expressed.

The functions of orexins and the architecture of orexin neurons are also highly conserved among mammalian species. On the other hand, orexin-like genes are not found in invertebrates, suggesting that the orexin system originated in early vertebrates.

## Evolution of Orexin Genes and Peptides

Thanks to genome research and previous molecular cloning studies, the amino acid sequences of orexins in several mammalian species (human, gorilla, golden monkey, baboon, gibbon, mouse, rat, pig, dog, camel, alpaca, seal, and dolphin) (Sakurai et al., 1998; Dyer et al., 1999; Peyron et al., 2000; Elbers et al., 2019), as well as reptiles (cobra and turtle) (Vonk et al., 2013), amphibians (*Xenopus laevis*) (Shibahara et al., 1999), birds (chicken, turkey, and finch) (Ohkubo et al., 2002), and fish (goldfish, zebrafish, cod, stickleback, medaka, pufferfish) (Kaslin and Panula, 2001), are currently available.

**FIGURE 1:**
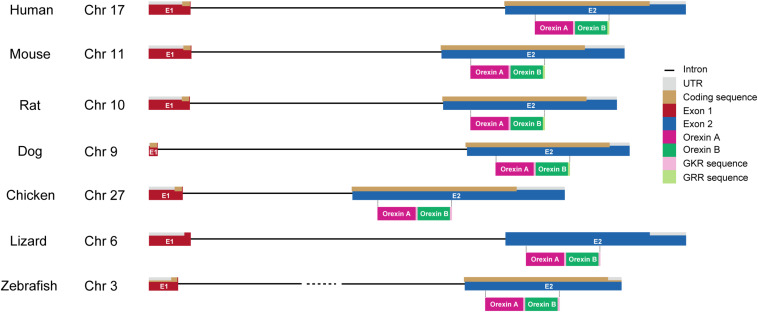
Gene structure of orexin in vertebrates. Chromosome numbers (Chr) in which the orexin gene is located are shown in each vertebrate. The drawing shows the length of gene structures of orexin including intron (black line), UTR (gray), coding sequences (brown), exon 1 (E1; red), and exon 2 (E2; blue) in different species (human, mouse, rat, dog, chicken, lizard, and zebrafish). Amino acid sequences of orexin A (magenta) and orexin B (dark green) with GKR (pink) and/or GRR (light green) amidation are also shown under their coding sequences.

**FIGURE 2:**
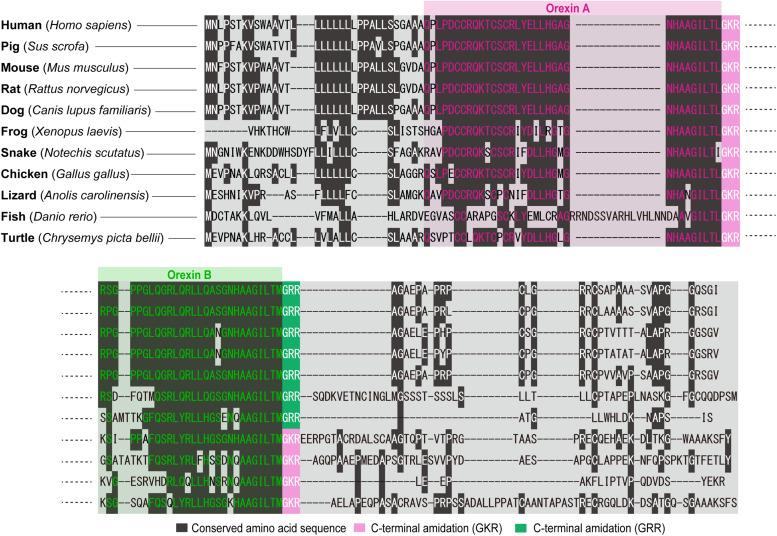
Overview of amino acid sequences of orexins in vertebrates. The cartoon shows the sequences of amino acids coding orexins A and B in different species (human: *Homo sapiens*; pig: *Sus scrofa*; mouse: *Mus musculus*; rat: *Rattus norvegicus*; dog: *Canis lupus familiaris*; frog: *Xenopus laevis*; snake: *Notechis scutatus*; chicken: *Gallus gallus*; lizard: *Anolis carolinensis*; fish: *Danio rerio*; and turtle: *Chrysemys picta bellii*). Conserved amino acid sequence (dark gray), C-terminal amidation (GKR, pink), and C-terminal amidation (GRR, green) are highlighted. Amino acid sequences are aligned by ClustalW algorithm using MEGA software (Stecher et al., 2020).

The genes encoding *prepro-orexin* show highly conserved loci throughout vertebrate evolution, including the two-exon structure, with a larger exon 2, which includes sequences encoding orexins A and B. Exon 1 generally contains 5’-UTR and part of the signal sequence. In the *prepro-orexin* sequence, orexin A sequences are directly preceded by signal peptides. Both mature peptides are followed by a putative consensus motif for C-terminal amidation (G-R/K-R/K) (Figures 1, 2). The C-terminal regions of *prepro-orexin* sequences following the orexin sequence are variable among species, suggesting that no other functional peptides are encoded in the region. Rat orexin A has been purified, and its structure analyzed by peptide sequencing and mass spec analyses. It has a 33-amino-acid peptide sequence with two intrachain disulfide bridges formed by four cysteine residues (C6–C12 and C7–C14), an N-terminal glutamate residue, and C-terminal amidation. The primary sequence of mammalian orexin A was shown to be modified to have an N-terminal pyroglutamyl residue and C-terminal amidation. The structure of orexin A is completely conserved among all mammalian species thus far identified. Mammalian orexin B is a 28-amino-acid linear peptide not having disulfide bridges and has minor amino acid differences among mammalian species. In particular, the second amino acid residue is P or S depending on the species (Figure 2).

Non-mammalian orexins are also very similar to mammalian orexin A but show more variations than those in mammals. Generally, there is no N-terminal pyroglutamylation in the orexin A structure due to lack of an N-terminal glutamate residue.

Bird (chicken, turkey, and finch) orexin A sequences contain 34 amino-acid residues and have two intra-disulfide bridges (C7–C13 and C8–C15), while orexin B sequences are 28-amino-acid linear peptides.

In reptiles, turtle (*Terrapene carolina triunguis*) orexin A is 34 amino acids long and is predicted to have a similar structure to that of mammalian orexin A, including two intra-disulfide bridges (C7–C13 and C8–C15), while orexin B is 28 amino acids long, which is the same as mammalian orexin B. C-terminal residues (L and M for orexins A and B, respectively) are likely to be amidated as mammalian orexin B, being predicted from glycine residues preceding dibasic pairs of amino acids. Snake (cobra) orexin A is 32 amino acids long and has two intra-disulfide bridges (C5–C11 and C6–C13).

In amphibians, *Xenopus laevis* orexin A is a 31-amino-acid-residue peptide and has six-amino-acid substitutions when compared with human orexin A (Shibahara et al., 1999). *Xenopus* orexin A does not have an N-terminal pyroglutamate residue either. The relative positions of the four cysteine residues (positions 4, 5, 10, and 12) are well conserved, and it is predicted to form two intrachain disulfide bonds (C4–C10 and C5–C12).

Teleost orexin A sequences are generally much longer than mammalian orexin A. For example, *Fugu* orexin A has 43 amino acid residues. Goldfish and zebrafish orexin A have 47 amino acid residues, and cod orexin A has 50 amino acid residues. These longer sequences are due to the existence of an additional sequence between residues 24 and 25 (Kaslin and Panula, 2001; Xu and Volkoff, 2007). The inserted sequences are non-detrimental to orexin activity (Kaslin and Panula, 2001). Because teleost orexin A does not have C12, it does not have a disulfide bond between C6 and C12 as found in mammalian orexin A, although it is likely to form another disulfide bridge with a cysteine positioned at 21. Teleost orexin B consists of 28 amino acid residues, which is the same as mammals’ and other species’ orexin B (Kaslin and Panula, 2001), with the exception of cod orexin B (29 amino acids) (Xu and Volkoff, 2007).

Overall, structures of orexins are exceptionally well conserved in the animal kingdom from fish to mammalian species. Teleost orexin A and human orexin A still have over 52% amino acid identity. The lengths and structures of orexin B are well conserved among species as compared with orexin A, suggesting that orexin B might be a prototype of orexin peptides.

## Evolution of Orexin Receptors

In mammals, there are two orexin receptor subtypes, OX1R and OX2R, both of which are members of the class B GPCRs. Orexin A shows similar affinity to both OX1R and OX2R, while orexin B shows higher affinity to OX2R over OX1R. The human OX1R and OX2R genes are located on chromosomes 1 and 6, respectively. Human OX1R and OX2R share 63.5% amino acid identity. They have also similarity to several other peptide receptors. For example, human neuropeptide FF receptor 1 shows 25.1 and 31.2% amino acid identity to OX1R and OX2R, respectively (Sakurai et al., 1998).

OX1R is exclusively found in mammalian species and is thought to have evolved from ancestral OX2R, presumably through gene duplication events during the evolution of early mammals. OX2R is present in all vertebrate genomes, suggesting that OX2R is the ancestral form of orexin receptors. The chromosomal localization of these receptors also suggests that OX1R is a product of a relatively recent gene duplication event from OX2R. The flanking genes of OX2R (FAM83B and GFRAL) are also well conserved in all known mammalian species. While TINAGL1 and PEF1 genes are in close proximity to the mammalian OX1R gene, they are not found in paralogous regions in non-mammalian genomes.

Because OX1R was emerged later than OX2R phylogenetically, it seems to play more complex physiological roles. We previously found that OX1R-deficient mice show anxiety-like behavior (Abbas et al., 2015). We also showed that OX1R in noradrenaline neurons in the locus coeruleus (LC) plays a role in the expression and/or consolidation of cued fear memory by exciting these neurons that send innervations to the lateral amygdala (Soya et al., 2013). Furthermore, this pathway was also involved in generalization of fear memory (Soya et al., 2017; Soya and Sakurai, 2018). OX1R was also shown to be involved in an increase of response to conditioned cues to activate motivational responses in rats (Sharf et al., 2010; Bentzley and Aston-jones, 2015) and in reward-based feeding (Kakizaki et al., 2019). These observations suggest that OX1R plays a role in emotive and motivational functions in mammals.

## Evolution of Orexin Neuronal System

Orexin neurons are localized in the LHA and adjacent regions, including the dorsomedial and posterior hypothalamus, in all mammalian species (Peyron et al., 1998; Date et al., 1999; Nambu et al., 1999). These neurons send widespread axonal projections to all over the brain except the cerebellum, with especially abundant projections to monoaminergic nuclei in the brain stem. In mammals, orexin neurons receive and integrate internal and external information and regulate the autonomic and neuroendocrine systems to stabilize arousal and behavior accordingly. The hypothalamus is the main region in which orexinergic neurons are localized in many species among vertebrates. In the chicken, orexin neurons are also exclusively found in the hypothalamus, including the paraventricular hypothalamic nucleus (PVN) and LHA (Miranda et al., 2013). In reptiles, orexin neurons are also found in the hypothalamus. In the *Pseudemys scripta elegans* (turtle) and *Anolis carolinensis* (lizard), these neurons are localized in the PVN, while in the *Gekko gecko* (lizard), these cells are found in the dorsomedial nuclei (DMH) (Farrell et al., 2003; Domínguez et al., 2010).

**FIGURE 3:**
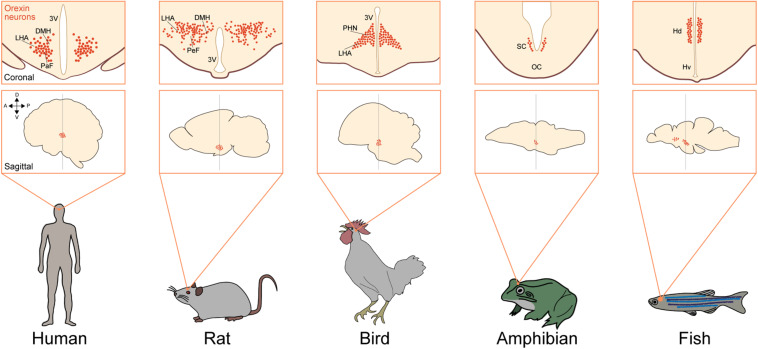
Neuronal system of orexin is highly conserved in vertebrates. Upper squares show a coronal view of the brain focusing on orexin neurons (red dots) in different species [human (Peyron et al., 2000), rat (Nambu et al., 1999), bird (Ohkubo et al., 2002), amphibian (López et al., 2009), and fish (Appelbaum et al., 2009)]. Lower squares show a sagittal view of the brain focusing on the populations of orexin neurons in different species. Arrows show the anatomical orientation (A, anterior; P, posterior; D, dorsal; and V, ventral). DMH, dorsomedial nuclei of hypothalamus; LHA, lateral hypothalamic area; PaF, parafornical nucleus; 3V, third ventricle; PeF, perifornical hypothalamus; PHN, periventricular hypothalamic nucleus; VM, ventromedial thalamic nucleus; SC, suprachiasmatic nucleus; OC, optic chiasm; Hd, dorsal zone of periventricular hypothalamus; Hv, ventral zone of periventricular hypothalamus.

In amphibians, orexins are found in the hypothalamus but are widely distributed in several regions outside the hypothalamus. These cells are localized especially in the suprachiasmatic nucleus (SCN) and to a lesser extent in the preoptic area (POA) and tuberal region in anurans, urodeles, and gymnophionans (Figure 3). Orexin-immunoreactive fibers innervate the whole-brain region, especially the POA (Shibahara et al., 1999; Galas et al., 2001; Singletary et al., 2005; Suzuki et al., 2008; López et al., 2009).

In fish, distribution of orexin neurons is more variable among species. For example, orexin neurons are localized in the POA and SCN in the lungfish and zebrafish. These neurons are found in the nucleus posterioris periventricularis (NPPv) in the medaka and in the NPPv and nucleus lateralis tuberis (NLT) in the goldfish. In zebrafish, orexin neurons are also localized in the dorsal part of the hypothalamus (Figure 3). Orexin fibers were shown to innervate the monoaminergic nuclei, including the dorsal raphe (DR), LC, mesopontine-like area, dopaminergic clusters, and histaminergic neurons in the tuberomammillary nucleus (TMN), showing resemblance to the mammalian orexin system (Kaslin and Panula, 2001; Huesa et al., 2005; Nakamachi et al., 2006; Amiya et al., 2007; Kojima et al., 2009). These findings suggest that orexin neurons are basically found in the hypothalamus and send rich projections to monoaminergic neurons. This basic structure is conserved in vertebrate evolution.

## Evolution of Orexin Functions

In mammals, orexin neurons receive and integrate internal and external information and regulate the autonomic and neuroendocrine systems during performance of various purposeful activities that require arousal. In this section, we discuss the evolution of the orexin system, especially focusing on two main functions of orexin, i.e., regulation of feeding behavior and wakefulness.

### Food Intake and Body Weight Regulation

Orexins were initially reported as factors that regulate feeding behavior, mainly because orexin neurons are distributed within the LHA (and adjacent regions), which is known to be implicated in the regulation of feeding behavior (Sakurai et al., 1998). An orexigenic effect of intracerebroventricular (ICV) administration of orexins A and B in rats was first reported in 1998, and this effect has been subsequently confirmed in many species, including mammals and other species, including fish (Nakamachi et al., 2006). Importantly, orexin signaling increased not only food intake but also energy expenditure, and an increase in the overall orexin tone generally results in decreased body weight (Funato et al., 2009). Likewise, narcoleptic mice, which lack orexin signaling, show mild obesity, especially when fed a high-fat diet.

The orexin system may contribute to the regulation of energy homeostasis by integrating information regarding metabolic state and regulating sleep/wakefulness states in order to support feeding behavior (Sakurai et al., 1998; Haynes et al., 2000; Yamada et al., 2000; Yamanaka et al., 2003; Funato et al., 2009; Sakurai, 2014). Indeed, mice lacking orexin neurons do not show an increase in wakefulness or locomotor activity in response to starvation, unlike wild-type mice (Yamanaka et al., 2003). Moreover, *prepro-orexin* mRNA is upregulated in fasted animals, and several studies report that the firing rate of orexin neurons is influenced by glucose, triglycerides, and amino acids (Chang et al., 2004; Burdakov et al., 2005; Karnani et al., 2011). Furthermore, orexin neurons are directly inhibited by leptin and excited by ghrelin and are innervated by neurons in the arcuate nucleus, which is the primary sensor for plasma leptin level (Elias et al., 1998; Yamanaka et al., 2003). Together, these observations suggest that orexin neurons sense the animal’s metabolic and nutritional status and integrate this information in order to evoke arousal necessary to promote food-seeking behavior in response to a negative energy balance. The precise mechanisms by which orexins regulate feeding behavior are detailed in our previous review papers (Sakurai, 2007, 2014).

Other than in mammalian species, the roles of orexins in the regulation of food intake are not very clear. In birds, orexin neurons and fibers are present in the PVN and LHA. This distribution is similar to that of mammalian orexin neurons. However, mammalian orexins did not increase food intake in birds (chicken and pigeon) (Furuse et al., 1999; da Silva et al., 2008; Katayama et al., 2010). However, in these studies, orexins were administered during the light period, which is the active period for birds, when orexin neuronal activity might be highest in the day. This may explain why additional orexin activity did not increase food intake. In fact, when administered in the dark period, orexins do not increase food intake even in rodents, which are nocturnal animals. Also, studies using avian orexin peptides, which are structurally different from mammalian orexins, are necessary to confirm whether orexins play roles in regulation of feeding behavior.

There are a substantial number of reports about the involvement of orexins in the regulation of feeding behavior in fish. ICV injection of human orexins increased food intake in goldfish (Volkoff et al., 1999; Nakamachi et al., 2006), and fasting increased *prepro-orexin* mRNA levels in zebrafish, as in mammals (Novak et al., 2005). Like mammals, a reciprocal relationship between orexins and ghrelin was reported in fish. Ghrelin increased the expression of *prepro-orexin* mRNA in the goldfish diencephalon when administered ICV and vice versa (Miura et al., 2007). Both neuropeptide Y (NPY)- and ghrelin-induced food intake were completely inhibited by application of an orexin receptor antagonist (Miura et al., 2007). The relationship between orexin and NPY was also shown by the colocalization of these peptides in the NPPv (Miura et al., 2007).

In other species, the roles of orexins in the regulation of feeding behavior have not been clear so far.

### Sleep/Wakefulness State Regulation

The involvement of orexins in the regulation of sleep/wakefulness states in mammals has been extensively discussed in detail in many review articles (Sakurai, 2007, 2014). The finding that orexin deficiency caused narcolepsy in humans and other mammalian species, like mice, rats, and dogs, clearly indicates that orexin plays an important role in the maintenance of long, consolidated wakefulness in mammals.

Other than in mammals, the roles of orexins in the regulation of sleep/wakefulness states are not very clear, but human orexin A induced dose-dependent arousal- and alertness-promoting behavioral effects in birds (chicken and pigeon) when administered ICV, along with a decrease in duration of sleep-like postures (da Silva et al., 2008; Katayama et al., 2010).

In zebrafish, sleep is usually defined solely by behavioral criteria based on periods of quiescence associated with a specific posture (Hendricks et al., 2000; Tononi, 2000; Zhdanova et al., 2001; Raizen et al., 2008). Genetic ablation of orexin neurons demonstrated an increase in sleep time and sleep/wakefulness transition in the daytime, with no effect on basal locomotor activity in zebrafish (Elbaz et al., 2012). Consistently, global overexpression of the orexin gene by an inducible heat-shock promoter showed an increase in wakefulness, defined by active behavior (Prober et al., 2006). A recent study showed that orexin-induced arousal is regulated via noradrenaline signaling in zebrafish (Singh et al., 2015). On the contrary, zebrafish orexins were reported to be involved in melatonin production in the pineal gland during the dark time, to regulate sleep consolidation (Appelbaum et al., 2009). Evolutional sleep loss was reported in the Mexican cavefish, *Astyanax mexicanus*, depending on their ecological conditions. The populations living in caves are blind, and their sleep time is shorter than that of other eyed populations living in surface rivers (Duboué et al., 2011). A recent study reported that the mechanism of this difference could stem from genetic and neuronal changes of orexins in the hypothalamus (Jaggard et al., 2018).

### Other Functions

Arousal responses are tightly associated with the physiological responses elicited by salient emotional stimuli. Several studies have suggested the involvement of orexins in regulating emotional behavior. A possible role of orexins in panic disorders has been reported in human and animal studies (Johnson et al., 2010). The LHA is known as the “defense area,” and orexins have functions to increase cardiovascular activity and stress response (Wilson et al., 2001; Sakamoto et al., 2004; Winsky-Sommerer et al., 2004; Zhang et al., 2009; Sakurai, 2014). Orexin neurons receive dense innervations from limbic structures like the BNST and the amygdala (González et al., 2016; Saito et al., 2018), suggesting that orexins regulate autonomic/neuroendocrine functions in response to emotional stimuli in mammals.

Other than in mammals, the roles of orexins in the regulation of emotional behavior, autonomic function, and neuroendocrine functions have not been clear, but psychomotor activity in goldfish was affected by an ICV injection of orexin A, suggesting an anxiogenic function of orexins, and this effect was abolished by injection of an OX1R antagonist (SB334867) (Nakamachi et al., 2014).

## Conclusion

Orexins play a highly important role in the regulation of sleep/wakefulness states in mammals. They are thought to be especially important for consolidation of wakefulness. Orexin deficiency results in narcolepsy, which is characterized by the inability to maintain long consolidated wakefulness, which is necessary to support any purposeful behaviors. Phylogenetically, orexins first appeared in vertebrates. They seem to be involved in the maintenance of wakefulness to pursue active motivated behavior in both mammals and other lower species. Even in mice, orexin neurons are relatively quiescent during quiet wakefulness, while they are active during active wakefulness, which accompanies purposeful behavior. This suggests that orexins are closely related to functions that support active behavior and consistently play a role as behavioral modulators among a wide range of species.

## Author Contributions

SS and TS contributed to writing and making the figures in this article and approved its submission for publication. Both authors contributed to the article and approved the submitted version.

## Conflict of Interest

The authors declare that the research was conducted in the absence of any commercial or financial relationships that could be construed as a potential conflict of interest.

## Abbreviations

A: anterior
BNST: bed nucleus of the stria terminalis
Chr: chromosome number
D: dorsal
DMH: dorsomedial nuclei of hypothalamus
DR: dorsal raphe
GPCR: G-protein -coupled receptor
Hd: dorsal zone of periventricular hypothalamus
Hv: ventral zone of periventricular hypothalamus
ICV: intracerebroventricular
LC: locus coeruleus
LHA: lateral hypothalamic area
NAc: nucleus accumbens
NLT: nucleus lateralis tuberis
NPPv: nucleus posterioris periventricularis
NPY: neuropeptide Y
OC: optic chiasm
OX1R: orexin 1 receptor
OX2R: orexin 2 receptor
P: posterior
PaF: parafornical nucleus
PeF: perifornical hypothalamus
PHN: periventricular hypothalamic nucleus
POA: preoptic area
PVN: paraventricular nucleus
SCN: suprachiasmatic nucleus
TMN: tuberomammillary nucleus
V: ventral
3V: third ventricle
VM: ventromedial thalamic nucleus.

## References

[B1] Abbas M. G. Shoji H. Soya S. Hondo M. Miyakawa T. Sakurai T. (2015). Comprehensive behavioral analysis of Ox1r-/- mice showed implication of orexin receptor-1 in mood, anxiety and social behavior. *Front. Behav. Neurosci.* 9:324. 10.3389/fnbeh.2015.00324 26696848 PMC4674555

[B2] Amiya N. Amano M. Oka Y. Iigo M. Takahashi A. Yamamori K. (2007). Immunohistochemical localization of orexin/hypocretin-like immunoreactive peptides and melanin-concentrating hormone in the brain and pituitary of medaka. *Neurosci. Lett.* 427 16–21. 10.1016/j.neulet.2007.07.043 17935885

[B3] Appelbaum L. Wang G. X. Maro G. S. Mori R. Tovin A. Marin W. (2009). Sleep – wake regulation and hypocretin – melatonin interaction in zebrafish. *Proc. Natl. Acad. Sci. U.S.A.* 10 1–6. 10.1073/pnas.906637106 19966231 PMC2799794

[B4] Bentzley B. S. Aston-jones G. (2015). Orexin-1 receptor signaling increases motivation for cocaine-associated cues. *Eur. J. Neurosci.* 41 1149–1156. 10.1111/ejn.12866 25754681 PMC4420694

[B5] Burdakov D. Gerasimenko O. Verkhratsky A. (2005). Physiological changes in glucose differentially modulate the excitability of hypothalamic melanin-concentrating hormone and orexin neurons in situ. *J. Neurosci.* 25 2429–2433. 10.1523/JNEUROSCI.4925-04.2005 15745970 PMC6726089

[B6] Chang G. Q. Karatayev O. Davydova Z. Leibowitz S. F. (2004). Circulating triglycerides impact on orexigenic peptides and neuronal activity in hypothalamus. *Endocrinology* 145 3904–3912. 10.1210/en.2003-1582 15117877

[B7] Chemelli R. M. Willie J. T. Sinton C. M. Elmquist J. K. Scammell T. Lee C. (1999). Narcolepsy in orexin knockout mice: molecular genetics of sleep regulation. *Cell* 98 437–451. 10.1016/S0092-8674(00)81973-X 10481909

[B8] da Silva E. S. dos Santos T. V. Hoeller A. A. dos Santos T. S. Pereira G. V. Meneghelli C. (2008). Behavioral and metabolic effects of central injections of orexins/hypocretins in pigeons (*Columba livia*). *Regul. Pept.* 147 9–18. 10.1016/j.regpep.2007.12.003 18234360

[B9] Date Y. Ueta Y. Yamashita H. Yamaguchi H. Matsukura S. Kangawa K. (1999). Orexins, orexigenic hypothalamic peptides, interact with autonomic, neuroendocrine and neuroregulatory systems. *Proc. Natl. Acad. Sci. U.S.A.* 96 748–753. 10.1073/pnas.96.2.748 9892705 PMC15208

[B10] De Lecea L. Kilduff T. S. Peyron C. Gao X. B. Foye P. E. Danielson P. E. (1998). The hypocretins: hypothalamus-specific peptides with neuroexcitatory activity. *Proc. Natl. Acad. Sci. U.S.A.* 95 322–327. 10.1073/pnas.95.1.322 9419374 PMC18213

[B11] Domínguez L. Morona R. Joven A. González A. López J. M. (2010). Immunohistochemical localization of orexins (hypocretins) in the brain of reptiles and its relation to monoaminergic systems. *J. Chem. Neuroanat.* 39 20–34. 10.1016/j.jchemneu.2009.07.007 19665547

[B12] Duboué E. R. Keene A. C. Borowsky R. L. (2011). Evolutionary convergence on sleep loss in cavefish populations. *Curr. Biol.* 21 671–676. 10.1016/j.cub.2011.03.020 21474315

[B13] Dyer C. J. Touchette K. J. Carroll J. A. Allee G. L. Matteri R. L. (1999). Cloning of porcine prepro-orexin cDNA and effects of an intramuscular injection of synthetic porcine orexin-B on feed intake in young pigs. *Domest. Anim. Endocrinol.* 16 145–148. 10.1016/S0739-7240(99)00011-19 10343916

[B14] Elbaz I. Yelin-Bekerman L. Nicenboim J. Vatine G. Appelbaum L. (2012). Genetic ablation of hypocretin neurons alters behavioral state transitions in zebrafish. *J. Neurosci.* 32 12961–12972. 10.1523/JNEUROSCI.1284-12.2012 22973020 PMC6703801

[B15] Elbers J. P. Rogers M. F. Perelman P. L. Proskuryakova A. A. Serdyukova N. A. Johnson W. E. (2019). Improving Illumina assemblies with Hi - C and long reads: an example with the North African dromedary. *Mol. Ecol. Resour.* 19 1015–1026. 10.1111/1755-0998.13020 30972949 PMC6618069

[B16] Elias C. F. Saper C. B. Maratos-Flier E. Tritos N. A. Lee C. Kelly J. (1998). Chemically defined projections linking the mediobasal hypothalamus and the lateral hypothalamic area. *J. Comp. Neurol.* 402 442–459. 10.1002/(sici)1096-9861(19981228)402:4<442::aid-cne2>3.0.co;2-r 9862320

[B17] Farrell W. J. Delville Y. Wilczynski W. (2003). Immunocytochemical localization of orexin in the brain of the green anole lizard (Anolis carolinensis). *Soc. Neur. Abstr.* 33:4.

[B18] Funato H. Tsai A. L. Willie J. T. Kisanuki Y. Williams S. C. Sakurai T. (2009). Enhanced orexin receptor-2 signaling prevents diet-induced obesity and improves leptin sensitivity. *Cell Metab.* 9 64–76. 10.1016/j.cmet.2008.10.010 19117547 PMC2630400

[B19] Furuse M. Ando R. Bungo T. Ao R. Shimojo M. Masuda Y. (1999). Intracerebroventricular injection of orexins does not stimulate food intake in neonatal chicks. *Br. Poult. Sci.* 40 698–700. 10.1080/00071669987115 10670685

[B20] Galas L. Vaudry H. Braun B. Van Den Pol A. N. De Lecea L. Sutcliffe J. G. (2001). Immunohistochemical localization and biochemical characterization of hypocretin/orexin-related peptides in the central nervous system of the frog Rana ridibunda. *J. Comp. Neurol.* 429 242–252. 10.1002/1096-9861(20000108)429:2<242::aid-cne5<3.0.co;2-z 11116217

[B21] González J. A. Iordanidou P. Strom M. Adamantidis A. Burdakov D. (2016). Awake dynamics and brain-wide direct inputs of hypothalamic MCH and orexin networks. *Nat. Commun.* 7:11395. 10.1038/ncomms11395 27102565 PMC4844703

[B22] Haynes A. C. Jackson B. Chapman H. Tadayyon M. Johns A. Porter R. A. (2000). A selective orexin-1 receptor antagonist reduces food consumption in male and female rats. *Regul. Pept.* 96 45–51. 10.1016/s0167-0115(00)00199-3 11102651

[B23] Hendricks J. C. Sehgal A. Pack A. I. (2000). The need for a simple animal model to understand sleep. *Prog. Neurobiol.* 61 339–351. 10.1016/S0301-0082(99)00048-49 10727779

[B24] Huesa G. van den Pol A. N. Finger T. E. (2005). Differential distribution of hypocretin (orexin) and melanin-concentrating hormone in the goldfish brain. *J. Comp. Neurol.* 488 476–491. 10.1002/cne.20610 15973685

[B25] Jaggard J. B. Stahl B. A. Lloyd E. Prober D. A. Duboue E. R. Keene A. C. (2018). Hypocretin underlies the evolution of sleep loss in the Mexican cavefish. *eLife* 7 1–22. 10.7554/eLife.32637 29405117 PMC5800846

[B26] Johnson P. L. Truitt W. Fitz S. D. Minick P. E. Dietrich A. Sanghani S. (2010). A key role for orexin in panic anxiety. *Nat Med* 16 111–115. 10.1038/nm.2075 20037593 PMC2832844

[B27] Kakizaki M. Kakizaki M. Tsuneoka Y. Takase K. Kim S. J. Choi J. (2019). Differential roles of each orexin receptor signaling in obesity differential roles of each orexin receptor signaling in obesity. *Iscience* 20 1–13. 10.1016/j.isci.2019.09.003 31546102 PMC6817686

[B28] Karnani M. M. Apergis-Schoute J. Adamantidis A. Jensen L. T. de Lecea L. Fugger L. (2011). Activation of central orexin/hypocretin neurons by dietary amino acids. *Neuron* 72 616–629. 10.1016/j.neuron.2011.08.027 22099463

[B29] Kaslin J. Panula P. (2001). Comparative anatomy of the histaminergic and other aminergic systems in zebrafish (*Danio rerio*). *J. Comp. Neurol.* 440 342–377. 10.1002/cne.1390 11745628

[B30] Katayama S. Hamasu K. Shigemi K. Cline M. A. Furuse M. (2010). Intracerebroventricular injection of orexin-A, but not orexin-B, induces arousal of layer-type neonatal chicks. *Comp. Biochem. Physiol. A Mol. Integr. Physiol.* 157 132–135. 10.1016/j.cbpa.2010.05.018 20595012

[B31] Kojima K. Kamijo M. Kageyama H. Uchiyama M. Shioda S. Matsuda K. (2009). Neuronal relationship between orexin-A- and neuropeptide Y-induced orexigenic actions in goldfish. *Neuropeptides* 43 63–71. 10.1016/j.npep.2009.01.004 19261328

[B32] Lin L. Faraco J. Li R. Kadotani H. Rogers W. Lin X. (1999). The Sleep Disorder Canine Narcolepsy Is Caused by a Mutation in the Hypocretin (Orexin) Receptor 2 Gene. *Cell* 98 365–376. 10.1016/S0092-8674(00)81965-81960 10458611

[B33] López J. M. Domínguez L. Moreno N. González A. (2009). Comparative immunohistochemical analysis of the distribution of orexins (hypocretins) in the brain of amphibians. *Peptides* 30 873–887. 10.1016/j.peptides.2009.01.013 19428764

[B34] Mieda M. Hasegawa E. Kisanuki Y. Y. Sinton C. M. Yanagisawa M. Sakurai T. (2011). Differential roles of orexin receptor-1 and -2 in the regulation of non-REM and REM sleep. *J. Neurosci.* 31 6518–6526. 10.1523/jneurosci.6506-10.2011 21525292 PMC3732784

[B35] Miranda B. Esposito V. De Girolamo P. Sharp P. J. Wilson P. W. Dunn I. C. (2013). Orexin in the chicken hypothalamus: immunocytochemical localisation and comparison of mRNA concentrations during the day and night, and after chronic food restriction. *Brain Res.* 1513 34–40. 10.1016/j.brainres.2013.03.036 23548597

[B36] Miura T. Maruyama K. Shimakura S. I. Kaiya H. Uchiyama M. Kangawa K. (2007). Regulation of food intake in the goldfish by interaction between ghrelin and orexin. *Peptides* 28 1207–1213. 10.1016/j.peptides.2007.03.023 17481778

[B37] Nakamachi T. Matsuda K. Maruyama K. Miura T. Uchiyama M. Funahashi H. (2006). Regulation by Orexin of Feeding Behaviour and Locomotor Activity in the Goldfish. *J. Neuroendocrinol.* 18 290–297. 10.1111/j.1365-2826.2006.01415.x 16503924

[B38] Nakamachi T. Shibata H. Sakashita A. Iinuma N. Wada K. Konno N. (2014). Orexin A enhances locomotor activity and induces anxiogenic-like action in the goldfish, Carassius auratus. *Horm. Behav.* 66 317–323. 10.1016/j.yhbeh.2014.06.004 24937437

[B39] Nambu T. Sakurai T. Mizukami K. Hosoya Y. Yanagisawa M. Goto K. (1999). Distribution of orexin neurons in the adult rat brain. *Brain Res.* 827 243–260. 10.1016/s0006-8993(99)01336-1330 10320718

[B40] Novak C. M. Jiang X. Wang C. Teske J. A. Kotz C. M. Levine J. A. (2005). Caloric restriction and physical activity in zebrafish (*Danio rerio*). *Neurosci. Lett.* 383 99–104. 10.1016/j.neulet.2005.03.048 15936519

[B41] Ohkubo T. Boswell T. Lumineau S. (2002). Molecular cloning of chicken prepro-orexin cDNA and preferential expression in the chicken hypothalamus. *Biochim. Biophys. Acta Gene Struct. Expr.* 1577 476–480. 10.1016/S0167-4781(02)00483-489 12359340

[B42] Peyron C. Faraco J. Rogers W. Ripley B. Overeem S. Charnay Y. (2000). A mutation in a case of early onset narcolepsy and a generalized absence of hypocretin peptides in human narcoleptic brains. *Nat. Med.* 6 991–997. 10.1038/79690 10973318

[B43] Peyron C. Tighe D. K. van den Pol A. N. de Lecea L. Heller H. C. Sutcliffe J. G. (1998). Neurons containing hypocretin (orexin) project to multiple neuronal systems. *J. Neurosci.* 18 9996–10015. 10.1523/JNEUROSCI.18-23-09996 9822755 PMC6793310

[B44] Prober D. A. Rihel J. Onah A. A. Sung R. J. Schier A. F. (2006). Hypocretin/orexin overexpression induces an insomnia-like phenotype in zebrafish. *J. Neurosci.* 26 13400–13410. 10.1523/JNEUROSCI.4332-06.2006 17182791 PMC6675014

[B45] Raizen D. M. Zimmerman J. E. Maycock M. H. Ta U. D. You Y. J. Sundaram M. V. (2008). Lethargus is a Caenorhabditis elegans sleep-like state. *Nature* 451 569–572. 10.1038/nature06535 18185515

[B46] Saito Y. Maejima T. Nishitani M. Hasegawa E. Yanagawa Y. Mieda M. (2018). Monoamines inhibit GABAergic neurons in ventrolateral preoptic area that make direct synaptic connections to hypothalamic arousal neurons. *J. Neurosci.* 38 6366–6378. 10.1523/JNEUROSCI.2835-17.2018 29915137 PMC6596100

[B47] Sakamoto F. Yamada S. Ueta Y. (2004). Centrally administered orexin-A activates corticotropin-releasing factor-containing neurons in the hypothalamic paraventricular nucleus and central amygdaloid nucleus of rats1: possible involvement of central orexins on stress-activated central CRF neurons. *Regul. Pept.* 118 183–191. 10.1016/j.regpep.2003.12.014 15003835

[B48] Sakurai T. (2007). The neural circuit of orexin (hypocretin): maintaining sleep and wakefulness. *Nat. Rev. Neurosci.* 8 171–181. 10.1038/nrn2092 17299454

[B49] Sakurai T. (2014). The role of orexin in motivated behaviours. *Nat. Rev. Neurosci.* 15 719–731. 10.1038/nrn3837 25301357

[B50] Sakurai T. Amemiya A. Ishii M. Matsuzaki I. Chemelli R. M. Tanaka H. (1998). Orexins and orexin receptors: a family of hypothalamic neuropeptides and G protein-coupled receptors that regulate feeding behavior. *Cell* 92 573–585. 10.1016/s0092-8674(00)80949-80946 9491897

[B51] Sharf R. Sarhan M. Brayton C. E. Guarnieri D. J. Taylor J. R. Dileone R. J. (2010). Orexin signaling via the Orexin 1 receptor mediates operant responding for food reinforcement. *Biol. Psychiatry* 67 753–760. 10.1016/j.biopsych.2009.12.035 20189166 PMC2849869

[B52] Shibahara M. Sakurai T. Nambu T. Takenouchi T. Iwaasa H. Egashira S. I. (1999). Structure, tissue distribution, and pharmacological characterization of Xenopus orexins. *Peptides* 20 1169–1176. 10.1016/S0196-9781(99)00120-125 10573288

[B53] Singh C. Oikonomou G. Prober D. A. Adamantidis A. Zhang F. Aravanis A. (2015). Norepinephrine is required to promote wakefulness and for hypocretin-induced arousal in zebrafish. *eLife* 4:e07000. 10.7554/eLife.07000 26374985 PMC4606453

[B54] Singletary K. G. Delville Y. Farrell W. J. Wilczynski W. (2005). Distribution of orexin/hypocretin immunoreactivity in the nervous system of the green Treefrog. *Hyla cinerea*. *Brain Res.* 1041 231–236. 10.1016/j.brainres.2005.01.095 15829232

[B55] Soya S. Sakurai T. (2018). Orexin as a modulator of fear-related behavior: hypothalamic control of noradrenaline circuit. *Brain Res.* 1731:146037. 10.1016/j.brainres.2018.11.032 30481504

[B56] Soya S. Shoji H. Hasegawa E. Hondo M. Miyakawa T. Yanagisawa M. (2013). Orexin receptor-1 in the locus coeruleus plays an important role in cue-dependent fear memory consolidation. *J. Neurosci.* 33 14549–14557. 10.1523/jneurosci.1130-13.2013 24005305 PMC6618384

[B57] Soya S. Takahashi T. M. Mchugh T. J. Maejima T. Herlitze S. Abe M. (2017). Orexin modulates behavioral fear expression through the locus coeruleus. *Nat. Commun.* 8 1–14. 10.1038/s41467-017-01782-z 29151577 PMC5694764

[B58] Stecher G. Tamura K. Kumar S. (2020). Molecular Evolutionary Genetics Analysis (MEGA) for macOS. *Mol. Biol. Evol.* 37 1237–1239. 10.1093/molbev/msz312 31904846 PMC7086165

[B59] Suzuki H. Kubo Y. Yamamoto T. (2008). Orexin-A immunoreactive cells and fibers in the central nervous system of the axolotl brain and their association with tyrosine hydroxylase and serotonin immunoreactive somata. *J. Chem. Neuroanat.* 35 295–305. 10.1016/j.jchemneu.2008.02.002 18378425

[B60] Thannickal T. C. Moore R. Y. Nienhuis R. Ramanathan L. Gulyani S. Aldrich M. (2000). Reduced number of hypocretin neurons in human narcolepsy. *Neuron* 27 469–474. 10.1016/s0896-6273(00)00058-1 11055430 PMC8760623

[B61] Tononi G. (2000). Correlates of sleep and waking in Drosophila melanogaster. *Science* 287 1834–1837. 10.1126/science.287.5459.1834 10710313

[B62] Volkoff H. Bjorklund J. M. Peter R. E. (1999). Stimulation of feeding behavior and food consumption in the goldfish, *Carassius auratus*, by orexin-A and orexin-B. *Brain Res.* 846 204–209. 10.1016/s0006-8993(99)02052-2051 10556637

[B63] Vonk F. J. Casewell N. R. Henkel C. V. Heimberg A. M. Jansen H. J. Woods A. E. (2013). The king cobra genome reveals dynamic gene evolution and adaptation in the snake venom system. *Proc. Natl. Acad. Sci. U.S.A.* 110 20651–20656. 10.1073/pnas.1314702110 24297900 PMC3870661

[B64] Wilson S. Baker J. Jessop D. S. Harbuz M. S. (2001). Central Orexin-A Activates Hypothalamic-pituitary-adrenal axis and stimulates hypothalamic corticotropin releasing factor and arginine vasopressin neurones in conscious rats. *J. Neuroendocrinol.* 13 421–424. 10.1046/j.1365-2826.2001.00655.x 11328451

[B65] Winsky-Sommerer R. Yamanaka A. Diano S. Borok E. Roberts A. J. Sakurai T. (2004). Interaction between the corticotropin-releasing factor system and hypocretins (orexins): a novel circuit mediating stress response. *J. Neurosci.* 24 11439–11448. 10.1523/JNEUROSCI.3459-04.2004 15601950 PMC6730356

[B66] Xu M. Volkoff H. (2007). Molecular characterization of prepro-orexin in Atlantic cod (*Gadus morhua*): cloning, localization, developmental profile and role in food intake regulation. *Mol. Cell. Endocrinol.* 271 28–37. 10.1016/j.mce.2007.03.003 17434256

[B67] Yamada H. Okumura T. Motomura W. Kobayashi Y. Kohgo Y. (2000). Inhibition of food intake by central injection of anti-orexin antibody in fasted rats. *Biochem. Biophys. Res. Commun.* 531 527–531. 10.1006/bbrc.1999.1998 10631095

[B68] Yamanaka A. Beuckmann C. T. Willie J. T. Hara J. Tsujino N. Mieda M. (2003). Hypothalamic Orexin neurons regulate arousal according to energy balance in mice. *Neuron* 38 701–713. 10.1016/S0896-6273(03)00331-333 12797956

[B69] Zhang W. Zhang N. Sakurai T. Kuwaki T. (2009). Orexin neurons in the hypothalamus mediate cardiorespiratory responses induced by disinhibition of the amygdala and bed nucleus of the stria terminalis. *Brain Res.* 1262 25–37. 10.1016/j.brainres.2009.01.022 19368849

[B70] Zhdanova I. V. Wang S. Y. Leclair O. U. Danilova N. P. (2001). Melatonin promotes sleep-like state in zebrafish. *Brain Res.* 903 263–268. 10.1016/S0006-8993(01)02444-2441 11382414

